# Assembly and Function of Gonad-Specific Non-Membranous Organelles in *Drosophila* piRNA Biogenesis

**DOI:** 10.3390/ncrna5040052

**Published:** 2019-11-06

**Authors:** Shigeki Hirakata, Mikiko C. Siomi

**Affiliations:** Department of Biological Sciences, Graduate School of Science, The University of Tokyo, Tokyo 113-0032, Japan; hirakata@bs.s.u-tokyo.ac.jp

**Keywords:** PIWI, piRNA, transposon, Yb body, Flam body, Dot COM, nuage, mitochondrion, *Drosophila*, ovary

## Abstract

PIWI-interacting RNAs (piRNAs) are small non-coding RNAs that repress transposons in animal germlines. This protects the genome from the invasive DNA elements. piRNA pathway failures lead to DNA damage, gonadal development defects, and infertility. Thus, the piRNA pathway is indispensable for the continuation of animal life. piRNA-mediated transposon silencing occurs in both the nucleus and cytoplasm while piRNA biogenesis is a solely cytoplasmic event. piRNA production requires a number of proteins, the majority of which localize to non-membranous organelles that specifically appear in the gonads. Other piRNA factors are localized on outer mitochondrial membranes. In situ RNA hybridization experiments show that piRNA precursors are compartmentalized into other non-membranous organelles. In this review, we summarize recent findings about the function of these organelles in the *Drosophila* piRNA pathway by focusing on their assembly and function.

## 1. Introduction

piRNAs are 24–35-nucleotide (nt) long non-coding RNAs that specifically associate with members of the PIWI subclade of the Argonaute protein family in a stoichiometric manner [1,2,3,4,5,6,7]. The association between PIWI and piRNA produces the piRNA-induced silencing complex (piRISC), the core engine of piRNA-mediated transposon silencing.

piRNAs have been identified in a variety of animals, ranging from hydra, sea anemone, flies, fish, and mammals, including humans [8,9]. In these species, piRNAs are produced from long non-coding RNAs transcribed from piRNA clusters. piRNA clusters are intergenic elements rich in transposon remnants dedicated for piRNA production via complicated biogenesis machineries. piRNAs mostly have sequences that are antisense to those of transposon mRNAs. Therefore, piRNAs can target piRISCs to transposon mRNAs to induce transposon repression. While some transposons in animals belong to the same family (e.g., long terminal repeat-type) [10], their sequences are not always conserved across species [11]. Therefore, the sequences of piRNA clusters and piRNAs are not conserved in sequences among species.

The number of PIWI members also differ in different species. For instance, *Drosophila* has three PIWI members, Piwi, Aubergine (Aub), and Ago3, while humans have four PIWI members, PIWIL1 to PIWIL4. Even in one species, the piRNAs loaded onto individual PIWI members show unique traits. In *Drosophila*, piRNAs loaded onto Piwi and Aub are mostly antisense to transposon mRNAs, while Ago3-bound piRNAs show a bias for the sense strand. Piwi- and Aub-bound piRNAs preferably contain uracil at the 5′ end, while Ago3-bound piRNAs tend to have adenine at the 10th nucleotide from the 5′ end [12,13]. The length of piRNAs associated with each PIWI member also vary. Piwi-piRNAs are about 26 nt long while Aub- and Ago3-piRNAs are about 25 nt and 24 nt, respectively [12].

The sequences of Piwi- and Aub-bound piRNAs are often identical. However, upon piRNA loading Aub and Piwi are localized to the cytoplasm and nucleus, respectively [12,14,15]. How they repress transposons also differ, with Piwi silencing transposons transcriptionally and Aub (and Ago3) doing so post-transcriptionally [1,2,3,4,5,6,7]. Lack of either of Piwi or Aub causes infertility, indicating that other family members do not compensate for their loss-of-function [16,17,18,19,20].

As members of the AGO subfamily of Argonaute proteins, cytoplasmic PIWI members, such as *Drosophila* Aub and Ago3, possess Slicer endonuclease activity and implement transposon silencing post-transcriptionally. In contrast, nuclear PIWI members, such as *Drosophila* Piwi, silence transposons transcriptionally in collaboration with multiple co-factors by inducing DNA methylation, repressive histone modification, and heterochromatinization [1,2,3,4,5,6,7]. *Drosophila* has both nuclear and cytoplasmic PIWI members, and subsequently has both transcriptional and post-transcriptional transposon silencing. In contrast, only cytoplasmic PIWI members are expressed in silkworm, and they do not possess transcriptional transposon silencing mechanisms [21]. Species-specific sex differences in PIWI member expression have also been reported. Mice express three PIWI members, all of which are essential for male, but not of female, fertility [22,23,24]. *Drosophila* requires all PIWI members for both female and male gonadal development and fertility [16,17,18,19,20].

piRNA studies have been extensively performed in flies, nematodes, and mice [3,4,5,8]. Biochemical and bioinformatic progress have recently been made in *Drosophila* cultured cell systems consisting of ovary-originating cells, such as fGS/OSSs and OSCs [25,26]. Both of these systems have functioning piRNA pathways, and interference in these pathways de-silences transposons. In this review, we outline the current understanding of the piRNA pathway in *Drosophila*, and share recent findings on gonad-specific non-membranous organelles and mitochondria, all of which contribute to piRNA biogenesis in *Drosophila*.

## 2. The piRNA Pathway in *Drosophila* Ovaries: The Outline

**Figure 1:**
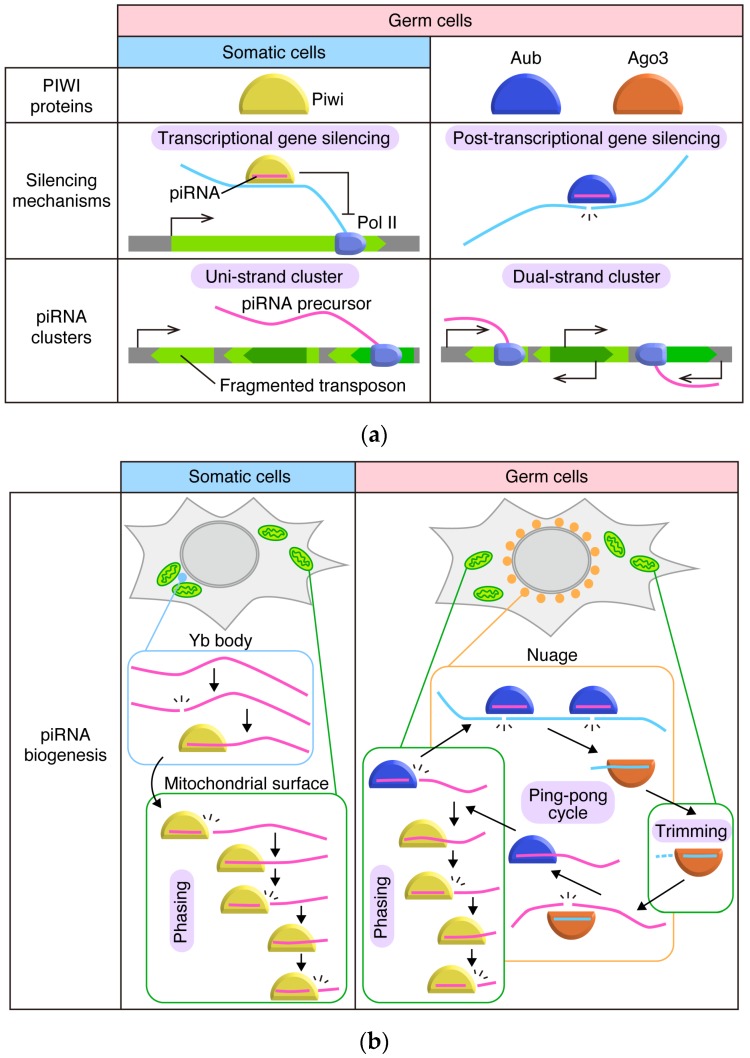
Comparison of *Drosophila* ovarian piRNA pathways in somatic cells and germ cells. (**a**) Comparison of PIWI proteins, silencing mechanisms, and piRNA clusters. Sole PIWI protein expressed in somatic cells, Piwi, is imported to nuclei once loaded with piRNA and represses transposon co-transcriptionally. In somatic cells, piRNA precursors are transcribed from uni-strand clusters, which are transcribed uni-directionally and produce transcripts harboring fragments of transposons in reverse orientation. In germ cells, Aub and Ago3 are expressed in addition to Piwi. Aub and Ago3 stay in the cytoplasm and cleave target transcripts using Slicer activities. piRNAs in germ cells are derived from both uni-strand and dual-strand clusters. Dual-strand clusters are transcribed from both strands of DNA, and transposon fragments are inserted in random orientations. (**b**) Comparison of piRNA biogenesis pathways. In somatic cells, dominant piRNA precursors, *flamenco*/*COM* (*flam*) transcripts, are selected and initially cleaved in Yb bodies, soma-specific perinuclear granules surrounded by mitochondria. Continuous cleavages of intermediate RNA on the outer membrane of mitochondria produce mature ‘phased’ piRNAs. In germ cells, reciprocal cleavage of target transcripts by Aub and Ago3 produce Ago3-bound and Aub-bound piRNAs, respectively. This cycle is called the ping-pong cycle. Through the ping-pong cycle, transposon transcripts (light blue) are cleaved and piRNAs are amplified. Cleavage of targets and loading of cleaved fragments onto PIWI proteins during the cycle occurs in germ-specific perinuclear granules called nuage. Maturation of Ago3-loaded piRNAs through trimming presumably occurs on the outer membrane of mitochondria. Loading of antisense RNA (magenta) on Aub triggers phased piRNA biogenesis on the mitochondrial outer membrane.

The *Drosophila* ovaries consist of germ (oocytes and nurse) cells and somatic (follicular) cells that surround the germ cells and produce eggshells [27,28]. The germ (nurse) cells express all PIWI members—Ago3, Aub, and Piwi—while ovarian somatic cells only express Piwi (Figure 1a) [12,14,29]. Aub and Ago3 are cytoplasmic and repress transposons post-transcriptionally [12,13]. Piwi is localized in the nucleus and represses transposons transcriptionally with co-factors, including Panoramix (Panx), Gtsf1, Nxf2, Nxt1/p15, Maelstrom (Mael), Eggless (Egg), histone linker H1, and HP1 [2,7,12,14,15,30,31,32,33,34,35,36,37,38,39,40,41,42,43,44,45,46,47].

In *Drosophila*, piRNA clusters are classified as either uni-strand or dual-strand depending on the directionality of transcription (Figure 1a) [2,3,4,7,8,12,48,49]. Uni-strand clusters are literally transcribed in one fixed direction while dual-strand clusters are transcribed in both directions. Ovarian somatic cells dominantly use uni-strand clusters, the representative of which is *flamenco*/*COM* (*flam*) [2,3,4,7,8,12,48,49]. In contrast, germ cells dominantly use dual-strand clusters for piRNA expression [2,3,4,7,8,12,48,49]. The most commonly expressed piRNA cluster in germ cells is *42AB.* The uni-strand clusters are transcribed by RNA polymerase II (Pol II) as regular protein-coding genes are and the RNA transcripts are exported to the cytoplasm by the system that regular mRNAs depend for their export (for details, see Section 3). In contrast, the dual-strand clusters require a quite unique system for their transcription, involving germ-specific factors such as Rhino, Deadlock, Cutoff, and Moonshiner [50,51,52,53,54,55]. The resultant RNA transcripts are then exported to the cytoplasm in an Nxf3- and Bootlegger-dependent manner [56,57].

A subset of protein coding-genes also give rise to piRNAs in addition to proteins. piRNAs are particularly produced from the 3′ untranslated region (3′ UTR) and are known as “genic piRNAs” [25,58]. The most studied of these is the *traffic jam* (*tj*) gene in ovarian somatic cells. Genic piRNAs, in general, are unrelated to transposon silencing. The *bona fide* targets of genic piRNAs remain elusive.

Transcription of the piRNA clusters takes place in the nucleus, but processing of cluster transcripts occurs in the cytoplasm and requires numerous factors (Figure 1b). Some factors participate in piRNA biogenesis in both germ and somatic cells, but others are cell type-specific [2,3,4,5,7,8,59]. In ovarian somatic cells, piRNA processing factors are localized to either perinuclear non-membranous granules, Yb bodies, or outer mitochondrial membranes. Female sterile (1) Yb (Yb), Armitage (Armi), Sister of Yb (SoYb), Vreteno (Vret), and Shutdown (Shu) are detected at Yb bodies, while Zucchini (Zuc), Gasz, Minotaur (Mino), and Daedalus (Daed) are detected on mitochondria (for details, see Section 4 and Section 5) [60,61,62,63,64,65,66,67,68]. Armi, Vret, and Zuc are also expressed in germ cells [48,63,67,68,69,70,71,72,73,74,75,76]. Germ-specific factors include Brother of Yb (BoYb), Spindle-E/Homeless (Spn-E), and Qin/Kumo (Qin) [64,77,78,79].

Yb bodies are not assembled in germ cells [60]. This is simply because the core factor of Yb body assembly, Yb, is not expressed in the cells. Germ cells form a similar type of non-membranous granules called the nuage (Section 6) [80,81]. Using RNA in situ hybridization, dotty structures called Dot COM/Flam bodies were visualized in ovarian somatic cells, where the *flam* RNA transcripts (i.e., flam-piRNA precursors) accumulate (Section 3) [82,83,84,85,86].

Upon piRISC assembly, Aub cleaves transposon mRNAs, which then serve as substrates for producing piRNAs for Ago3 (Figure 1b). Therefore, Ago3-loaded piRNAs are mostly parallel, or in sense orientation, to transposon mRNAs. Ago3 then cleaves transposon transcripts in the antisense orientation. piRNAs produced from this reaction assemble piRISCs with Aub [12,13]. This reciprocal cleavage by Aub and Ago3 in sense and antisense directions, respectively, keeps producing Ago3- and Aub-bound piRNAs [12,13]. At the same time, transposons are repressed post-transcriptionally. This system, widely known as the “ping-pong cycle”, is considered to be the coupled event of transposon repression and piRNA biogenesis [12,13]. The ping-pong cycle is germ cell-specific and occurs in the nuage in the cells (Section 6).

## 3. Flam Body/Dot COM: Dotty Structures Where piRNA Precursors Accumulate before Processing in Ovarian Somatic Cells

The *flam* piRNA cluster is the origin of transposon-targeting piRNAs in OSCs [12,20,25,48,87]. *flam* transcription represses a number of transposons [48,88,89,90]. However, the *flam* piRNA cluster is inactive in germ cells, suggesting that piRNA-dependent transposon silencing is distinct in somatic and germ cells. The *flam* locus has a single promoter and transcription is induced by the Cubitus interruptus transcription factor [31,91]. The resultant transcripts are 5′-capped, polyadenylated, and alternatively spliced [91], hallmarks of canonical Pol II-dependent RNA products.

**Figure 2:**
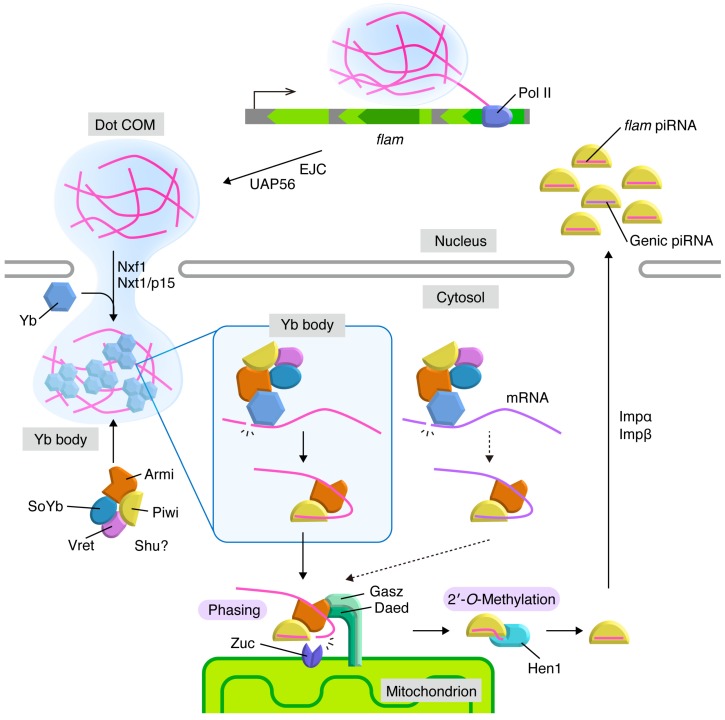
piRNA biogenesis pathway in *Drosophila* ovarian somatic cells. Transcripts of *flam* (magenta) generate Dot COM in the nucleus and are translocated to nuclear periphery in a manner dependent on Exon Junction Complex (EJC) and UAP56. Dot COM is exported to the cytoplasm by the Nxf1-Nxt1/p15 complex. In the cytoplasm, *flam* transcripts are processed into piRNA intermediates in Yb bodies. Yb and *flam* require each other for granularization in the cytoplasm. The Armi-Piwi-piRNA intermediate complex moves to the surface of the mitochondrial outer membrane, where phased piRNAs are produced in mature lengths by endoribonuclease Zuc. After Hen1-mediated 2′-*O*-methylations at the piRNA 3′ ends, piRISCs are imported into the nucleus, where they co-transcriptionally repress their targets. Some mRNAs (violet) are also processed into piRNA intermediates in the cytosol at low efficiency and generate genic piRNAs through phasing on mitochondria. The targets and functions of genic piRNAs are vague.

RNA fluorescent in situ hybridization (FISH) experiments show that *flam* RNAs accumulate at Dot COM [82,83,84,85]. Dot COM is found in both the nucleus and the cytoplasm (Figure 2). In the nucleus of wild-type ovaries, Dot COM is detected far from the *flam* locus. However, upon knockdown of components of the exon junction complex (EJC) and/or UAP56, known mRNA nuclear export factors, Dot COM is located proximal to the *flam* locus. This indicates that Dot COM might be assembled at the *flam* transcription site and move across the nuclei in an EJC- and UAP56-dependent manner [83]. The transcripts of other piRNA cluster components are also detected at Dot COM [82]. The mechanism of and/or requirement for Dot COM assembly remains elusive.

The export of *flam* transcripts depends on two mRNA export factors: Nxt1/p15 and Nxf1 [83]. Dot COM was detected to move across the nuclear pores to localize to the cytoplasm [83,84]. RNA FISH experiments determined that the *flam* RNAs accumulate in cytoplasmic structures called Flam bodies. Flam bodies are detected adjacent to Yb bodies and their formation is Yb-dependent [86]. It is highly likely that Flam bodies and cytoplasmic Dot COM are identical, but this has not yet been proven experimentally. Yb is a cytoplasmic protein, so formation of nuclear Dot COM may be Yb-independent.

## 4. Yb Bodies: Cytoplasmic Non-Membranous Organelles Where Transposon-Targeting piRNA Precursors Undergo Primary Processing in Ovarian Somatic Cells

Yb bodies are ovarian soma-specific, cytoplasmic non-membranous organelles. Yb bodies are located at perinuclear regions and there are only a few Yb bodies per cell [86]. Yb, Armi, SoYb, Vret, and Shu are components of Yb bodies (Figure 2) [60,61,62,63,64,65]. Knockdown of Yb, but not of the other components, causes the disappearance of Yb bodies. Therefore, Yb is considered the core component of Yb body assembly. The hierarchy of the other components in Yb body assembly is Armi to the SoYb-Vret heterodimer [61,62,63,64,65,92]. SoYb and Vret interact with and stabilize each other in vivo [92].

Yb has Helicase-C terminal (Hel-C), DEAD-box RNA helicase, and extended Tudor (eTud) domains [60,64,92]. The latter two domains confer RNA-binding activity to Yb, while the N-terminal Hel-C domain acts as a Yb self-assembly domain. The C-terminal eTud domain also plays a role in Yb-Armi binding [92]. Yb fails to interact with the SoYb-Vret heterodimer in the absence of Armi [92]. The eTud domain is also proposed to interact with the SoYb-Vret heterodimer through Armi.

Yb bodies are not formed if Yb is present in the cells but lacks RNA-binding activity [86]. This is consistent with the observation that the *flam* mutant ovaries form almost no Yb bodies [83,84]. Taken together, these results indicate that Yb binding to piRNA precursors, other than *flam,* is insufficient for Yb body assembly. Likewise, Yb mutants lacking self-association activity fail to assemble Yb bodies, even when the RNA-binding activity is maintained [92]. *flam* RNAs contain numerous Yb-binding sites, while genic piRNA sources have only a few 3′ UTR-restricted Yb-binding sites [93]. Based on these observations, it was inferred that Yb bodies are multivalent condensates formed by liquid–liquid phase separation (LLPS) requiring both Yb-*flam* RNAs and Yb-Yb associations. Live imaging demonstrated that Yb bodies in cultured OSCs are dynamic and fuse and divide repetitively, which are hallmarks of LLPS-driven condensates. Like P bodies, Yb bodies also show sensitivity to the LLPS disruptor, 1,6-hexanediol [92]. Thus, Yb bodies are LLPS-driven condensates in OSCs.

Yb bodies used to be known as the place of piRNA biogenesis in OSCs. However, recent studies show that Yb body formation is not absolutely mandatory for piRNA biogenesis. Indeed, non-transposon-targeting, genic piRNAs are produced in mutant OSCs as efficiently as in normal OSCs. In mutant OSCs ectopically expressing Hel-C lacking Yb instead of endogenous Yb, the Yb mutant bound *flam* RNAs and *tj* mRNAs to a similar extent. In contrast, wild-type Yb bound *flam* RNAs much more strongly than it did genic piRNA sources, including *tj* mRNAs [92]. Therefore, a more accurate definition of Yb bodies reads: Yb bodies are the place for producing transposon-targeting piRNAs in OSCs.

Armi has intrinsic, promiscuous RNA-binding activity [72,94]. In the presence of Yb, this “randomized” activity is hidden, but in the absence of Yb, Armi blindly induces the production of non-transposon-targeting piRNAs from bound RNAs [94]. Therefore, Armi has the activity to induce piRNA biogenesis in a Yb-independent manner. Indeed, tethering Armi to reporter RNAs in OSCs induces piRNA biogenesis from the reporter [69,94,95]. These findings support Armi being the facilitator of piRNA biogenesis in OSCs, with Yb being the selector of piRNA precursors through *cis-*elements embedded in the RNAs [93]. When both Yb and Armi proteins co-exist in vivo, transposon-targeting piRNAs are produced properly and transposons are efficiently silenced. Thus, the piRNA biogenesis pathway in OSCs takes full advantage of the characteristics of both Yb and Armi. The functions of SoYb, Vret, and Shu in somatic piRNA biogenesis have yet to be fully elucidated.

## 5. Mitochondria: Membranous Organelles that Serve as the Place for piRNA Maturation and Phased piRNA Biogenesis

Zuc, Daed, Mino, and Gasz contain transmembrane signals and localize to the outer membrane of mitochondria in OSCs (Figure 2). Lack of these factors reduces the levels of mature piRNAs and the aberrant accumulation of piRNA intermediates. This indicates that piRNA maturation occurs at the mitochondria [61,63,66,67,68]. Why piRNA factors congregate at the mitochondria to perform the final step in piRNA processing remains unknown.

Mitochondria surround Yb bodies in OSCs. This arrangement results in the concentration of piRNA factors and piRNA precursors in the vicinity of mitochondria and facilitates piRNA production [60,61,86]. However, there are only a few Yb bodies per cell and numerous mitochondria [86]. Moreover, there are high levels of piRNAs in OSCs. Therefore, it is very unlikely that only the fraction of Zuc that is located proximal to Yb bodies functions in piRNA processing. For the proportion of Zuc that is located far from Yb bodies to function in piRNA processing, RNA substrates would need to travel across the cytosol from Yb bodies to mitochondria. Recent studies show that the intermediates dynamically move from Yb bodies to mitochondria in association with Piwi and Armi [67,96]. In the complex, Piwi interacts with the 5′-phosphate group of the intermediates [96]. Armi also binds the RNAs but this interaction depends on Piwi [67]. Upon Zuc depletion, Armi aberrantly accumulated on the surface of mitochondria through Gasz. Additional knockdown of Gasz causes Armi to be stuck in Yb bodies [67,96]. Piwi behaves similarly, but to a lesser extent, and cannot stay longer on Gasz through Armi at the surface of mitochondria when Zuc function is abrogated [96]. The functions of Daed and Mino in OSCs remain elusive.

Armi localizes to Yb bodies through direct binding with Yb [92]. This action does not require Piwi [63]. However, Armi remains localized to Yb bodies in the absence of Piwi, indicating that the departure of Armi from Yb bodies requires Piwi [96]. Armi has the intrinsic ability to bind RNAs promiscuously. If Armi is released from Yb bodies, even without piRNA intermediates, Armi would randomly bind RNAs in the cytosol and blindly initiate piRNA production from these bound RNAs. To avoid this unwanted situation, OSCs have evolved a system to ensure that Armi is released from Yb bodies and is able to head to the mitochondria only upon binding to Piwi-piRNA intermediates.

On mitochondria, Zuc cleaves Piwi-bound piRNA intermediates to excise mature piRNAs. This cleavage determines the 3′ end of the mature piRNA and Piwi-piRISC release [74,75,97,98]. The cleavage also generates a novel 5′ end on the intermediate, which is then bound with apo-Piwi (Piwi devoid of piRNAs). In this manner, Zuc continuously and sequentially generates piRNAs, termed phased piRNAs, from one intermediate RNA towards its 3′ end [74,75]. To accomplish this Zuc reaction, the ATP hydrolysis-dependent 5′-to-3′ directional RNA helicase activity of Armi is required to relax RNA substrates and Armi has to undergo inter-organelle translocation [69,94]. Sometimes, Zuc produces piRNAs with a few extra bases at the 3′ end. These additional 3′ bases are removed by a 3′-to-5′ endonuclease Nibbler (Nbr) in germ cells [99,100,101]. In contrast, the additional bases are removed by unknown nuclease(s) in a manner dependent on a mitochondrial outer-membrane protein, Papi, in somatic cells [101]. The methyltransferase Hen1/Pimet 2′-*O*-methylates piRNAs at the 3′ ends to increase their stability in vivo [100,102,103].

In germ cells, SoYb, Zuc, Mino, Gasz, and Daed also localize to the outer mitochondrial membrane where they are involved in piRNA biogenesis [48,63,66,67,68,74,75,76]. The 3′ end of Aub-loaded piRNAs is determined by Zuc [101]. The remaining 3′ end fragment is funneled into phased piRNA biogenesis, from which Piwi-loaded piRNAs and, to a lesser extent, Aub-bound piRNAs are generated (Figure 1b) [74,75,101,104,105]. Translocation of piRNA intermediates from the nuage to mitochondria in germ cells also requires Armi, Gasz, and Daed [67,70,72].

Upon completion of piRNA processing, Piwi-piRISCs are promptly imported to the nucleus in an Impα/β dependent manner [106]. The nuclear import of Piwi is strictly regulated by Piwi-piRNA association, and apo-Piwi is not imported to the nucleus [61,62,63,65,66,67,68,96,107].

## 6. Nuage: The Place for piRNA Amplification and Post-Transcriptional Repression through the Ping-Pong Cycle in Germ Cells

In germ cells, the vast majority of piRNA factors are found in the nuage. This includes Vasa, Armi, Vret, Shu, BoYb, Krimper (Krimp), Spn-E, and Qin [64,65,67,68,69,70,71,72,73,77,78,79,107]. The hierarchy of these proteins in nuage formation (and/or localization) has primarily been studied genetically, but a solid conclusion has not yet been drawn. More thorough, comprehensive analyses are required to determine how the nuage is formed. These factors likely localize to different sets of nuage, and it is possible that the non-membranous structures in germ cells are not uniform [21,108,109,110].

Vasa is a germline-specific DEAD-box RNA helicase [12,111]. Recent investigations using cultured *Bombyx* ovary-derived germ cells show that Vasa uses the RNA helicase activity to release cleaved RNAs from Siwi, the silkworm Aub counterpart, upon Slicer-dependent cleavage. These cleaved RNAs are subsequently loaded onto Ago3 (in both fly and silkworm) and processed to mature piRNAs, facilitating the ping-pong cycle [21,112]. Siwi holds cleaved RNAs until Vasa releases them from the protein. This characteristic is unique to PIWI members, and is not observed for AGO members. This is reasonable because RNAs cleaved by AGO are further degraded in the cellular environment to complete target gene silencing, but RNAs cleaved by PIWI serve as the substrate for piRNA biogenesis (Section 2). The Vasa counterpart that works for Ago3 remains undetermined.

The human ortholog of Vasa, Ddx4, can form LLPS-driven condensates [113]. Fly genetic studies show that the nuage is not detected in vasa mutant ovaries [79,114,115]. Therefore, it is feasible that in *Drosophila* the nuage may be constructed through Vasa-driven phase separation.

The unstructured region, but not the helicase domain, of Vasa/Ddx4 is sufficient to induce phase separation [113]. Accordingly, nuage formation is thought to be RNA-independent. However, this notion is still controversial. UAP56, an export factor of piRNA precursors in germ cells, is localized to foci formed on dual-strand piRNA clusters. The nuclear foci are located near the cytoplasmic nuage, across the nuclear membrane [116]. The nuage disappeared in an UAP56 mutant that failed to localize to the cluster foci and did not export piRNA precursors [116]. Thus, nuclear export of piRNA precursors by UAP56 might trigger nuage formation.

Krimp, a Tudor domain-containing protein, plays an important role in the ping-pong cycle [48,79,117]. Krimp associates with Ago3 through symmetrically dimethylated arginines on Ago3 and brings it to the nuage, where Ago3 is loaded with piRNAs through the ping-pong cycle [79,108,118,119]. In the absence of Krimp, Ago3 becomes devoid of piRNAs and Aub initiates homotypic ping-pong cycles [118]. However, the efficiency of piRNA production through homotypic ping-pong is very low and leads to the eventual derepression of transposons, causing severe damages to the cells. Loss of Qin, another Tudor domain-containing protein, also results in Aub-Aub homotypic ping-pong in the nuage [77,120]. Under these conditions, Piwi is loaded with sense transcripts cleaved by Aub [105]. The heterotypic ping-pong cycle ensures that only antisense transcripts are being used for phased piRNA biogenesis and bearing Piwi-loaded piRNAs. OSCs also express Krimp, but it is sequestered in Krimp bodies, cytoplasmic granules that are distinct from Yb bodies. Interestingly, Krimp is dispensable for piRNA biogenesis in OSCs [65,118]. Why Krimp bodies form in OSCs remains unknown.

Studies have also been conducted to understand the functions of the other nuage-specific piRNA factors. However, the results of such studies largely remain vague. Investigations using OSCs and fGS/OSSs have greatly contributed to our understanding of the mechanisms underlying Yb body formation and other aspects in the piRNA pathway in ovarian somatic cells. Therefore, the establishment of cultured fly germ cells is eagerly awaited. Knockout of the lethal (3) malignant brain tumor [L(3)MBT], which encodes the transcriptional tumor repressor L(3)MBT, induced the ping-pong cycle in cultured OSCs. Many of the ping-pong factors, including Aub and Ago3, are not normally expressed in OSCs and the ping-pong cycle does not normally function in these cells [121]. Unlike original OSCs, OSC derivatives, termed Δmbt-OSCs, assembled nuage. These OSC derivatives might greatly contribute to our understanding of the mechanism of nuage formation.

## 7. Perspective

In this review, we have discussed the piRNA pathway in the *Drosophila* ovary. We have particularly focused on the functions of intracellular structures and their components and on the regulatory mechanisms of molecular migration between the structures. However, the functions of components in the structures are not completely understood. Furthermore, there are questions that remain to be addressed. For instance, how are nuclear Dot COM and the nuage assembled in vivo, and which of the nucleases is/are responsible for the first cleavage of the primary piRNA precursors? In addition, analyses focusing on post-translational modifications and temporal regulation during oogenesis are anticipated to further our understanding of the piRNA pathway. *Drosophila* ovaries, supported by our accumulating understanding of the piRNA pathway, the vast array of *Drosophila* genetic tools, the availability of cultured OSCs, and emerging technologies like genome editing, will continue to bring us novel insights about piRNAs. At the same time, information about piRNAs in testis and in other animals, and studies concerning the crosstalk between the piRNA pathway and other pathways, will inform the researchers investigating the *Drosophila* ovarian piRNA pathway. These studies will collaboratively approach the fundamental question of how animals tackle transposons with piRNAs.
